# Perceived Knowledge and Confidence for Providing Youth-Specific Type 1 Diabetes Exercise Recommendations amongst Pediatric Diabetes Healthcare Professionals: An International, Cross-Sectional, Online Survey

**DOI:** 10.1155/2023/8462291

**Published:** 2023-03-02

**Authors:** Paula Chinchilla, Klemen Dovc, Katarina Braune, Ananta Addala, Michael C. Riddell, Tiago Jeronimo Dos Santos, Dessi P. Zaharieva

**Affiliations:** ^1^London North West Hospitals, Department of Women & Children, London, UK; ^2^Department of Pediatric Endocrinology Diabetes and Metabolic Diseases, University Children's Hospital, Faculty of Medicine, University of Ljubljana, Ljubljana, Slovenia; ^3^Department of Pediatric Endocrinology and Diabetes, Charité-Universitätsmedizin Berlin, Berlin, Germany; ^4^Institute of Medical Informatics, Charité-Universitätsmedizin Berlin, Berlin, Germany; ^5^Berlin Institute of Health at Charité, Berlin, Germany; ^6^Division of Endocrinology, Department of Pediatrics, Stanford University, School of Medicine, Stanford, CA, USA; ^7^School of Kinesiology and Health Science, York University, Toronto, ON, Canada; ^8^LMC Manna Research, Toronto, ON, Canada; ^9^Pediatrics Unit, Vithas Almería, Instituto Hispalense de Pediatría, Almería, Andalusia, Spain

## Abstract

**Background:**

Managing glycemia around exercise is challenging for individuals with type 1 diabetes (T1D) and their healthcare professionals (HCP). We investigated HCP knowledge and confidence around exercise counseling for youth with T1D worldwide.

**Objective:**

To assess HCP familiarity with ISPAD Clinical Practice Consensus Guidelines and confidence to deliver recommendations about T1D and exercise.

**Methods:**

A new online survey was developed on strategies and competencies about exercise for youth with T1D, comprising of 64 questions, divided into eight different categories, assessing HCPs perceived exercise knowledge, confidence, training, and barriers to exercise counseling.

**Results:**

A total of 125 HCPs mean ± SD age 42 ± 8.2 years (74% female, 73% physicians) completed the survey. The ISPAD exercise guidelines were considered familiar to 68/125 (54%) of responders. Overall, 91/125 (73%) felt confident with giving recommendations about exercise with 47/125 (38%) recommending 45–60 mins/day of physical activity, while 16/125 (13%) recommended >60 mins/day. Several topics related to self-management around exercise were covered by most, but not all responders, and differences were observed in exercise content “confidence” and/or “competence” based on geographic location (*p* = 0.048). No differences in exercise recommendation dose, confidence, or familiarity with ISPAD guidelines were observed for age, sex, type of HCP, years in practice, or healthcare type.

**Conclusions:**

Exercise counseling for youth with T1D remains a challenge in most healthcare settings, globally. In general, the number of physically active minutes per week is under-prescribed for youth with T1D and many HCPs in various settings around the world feel that more professional education is needed to boost confidence around the education of several exercise-related topics.

## 1. Introduction

For youth with type 1 diabetes (T1D), maintaining glycemia in a targeted range during and after physical activity (PA or “exercise” for this research) is challenging. Although daily PA should be encouraged by healthcare professionals (HCP) for youth with T1D, a large percentage of the T1D youth and young adult population are not meeting the target guidelines [1–3]. According to a cross-sectional multicenter study of 18,028 adults (≥18 to <80 years of age) from Germany and Austria with T1D, ∼63% of adults with T1D are not physically active enough, as defined by the World Health Organization (WHO) [4]. Inactivity in youth, with or without disease, typically tracks into inactivity in adulthood, with further declines in daily activity over the young adult years, even in those who are relatively active as youth [5]. As such, encouraging and “prescribing” PA to youth with T1D should be a priority, with some additional emphasis on how to manage activity safely from a glycemic management perspective. According to the International Society for Pediatric and Adolescent Diabetes (ISPAD) exercise guidelines [6], all children and adolescents between 6 and 18 years of age should aim for at least 60 minutes of moderate-to-vigorous-intensity PA each day, a recommendation that is given for all youth globally by other authorities including the WHO [7]. Vigorous-intensity aerobic activities, like running, bicycle riding, and some individual and team sports, as well as those that strengthen muscle and bone, such as climbing, games of tug of war, and resistance exercises using body weight, should be incorporated into this 60 minutes of PA per day, at least three days a week for a variety of health and fitness reasons [2, 7, 8].

Regular PA in youth with T1D should be encouraged and supported by HCPs for many reasons, including overall psychosocial well-being, metabolic health, and cardiovascular benefits [9]. Prescribing “exercise” by HCP can help sedentary adults, with or without risk factors, overcome physical inactivity [10], but the effectiveness of this approach for sedentary youth with T1D is unclear. Some starting points for education around exercise management in T1D may include describing the types and amounts of PA that should be done, some nutritional support and general insulin dose adjustments based on the PA type and timing, and reasonable and individualized target glucose ranges for the various activities [1, 6, 11, 12]. However, common barriers to engaging in exercise for youth with T1D include the fear of hypoglycemia, a loss of glucose stability, low fitness levels, and insufficient or inadequate knowledge of strategies to prevent hypoglycemia [13–15].

There is limited data evaluating HCP knowledge about existing PA guidelines and management strategies for T1D, particularly between different countries. However, Knight and colleagues [16] demonstrated that over half of the HCPs in a United Kingdom (UK) survey reported a lack of knowledge and unclear guidelines as a barrier to sharing exercise-related education in a clinical setting. Lack of time in consultations, lack of confidence in the topic of exercise management in T1D, and insufficient funds to produce educational materials were noted as additional barriers. Inconsistencies are also apparent between the HCPs knowledge and confidence in advising “safe” exercise strategies for glucose self-management. Another UK study by Litchfield et al. [17] evaluated the patient and HCPs perspectives on the delivery of exercise education for adults with T1D and determined a need for more responsive and directed education programs for patients and providers internationally.

Our ISPAD international study explored pediatric diabetes HCPs knowledge and confidence around exercise for youth with T1D across five continents. An international survey was utilized to explore perceived knowledge and confidence to speak about various exercise topics, professional training received about exercise and diabetes, and barriers to speaking about exercise to youth with T1D.

## 2. Methods

An anonymous online survey about PA recommendation strategies and competencies for youth T1D was developed by our team (P.C. and K.D.) using Google forms (Google Inc. California). These questions were developed by a panel of experts in the field and people living with T1D from an ISPAD-related conference working group and distributed through an open weblink to members of ISPAD and past participants of Annual Meetings and training courses, which reach ∼2,300 HCPs. Newsletters, ISPAD Juniors in Educational Networking and International Research Opportunities: United Sessions (JENIOUS) social media platforms, and DiAthlete online community platforms were also used to gather this survey data. ISPAD JENIOUS connects HCPs below the age of 40 and is used to collaborate, promote, and develop projects for children and adolescents with diabetes. DiAthlete is a charity based in the UK that looks at delivering education about T1D through sports and games. The inclusion criteria were for respondents who work with children and adolescents with T1D.

A total of 64 survey questions were asked, consisting of yes/no responses, Likert scale (e.g., 1–5 scored and reverse scored for specific questions), and other open-ended questions. The survey questions were divided into the following categories: 1) demographic characteristics of the respondents (age, country, gender), ISPAD membership and awareness of exercise guidelines, place of work, years of practice, and profession; 2) advice and recommendations of exercise to children and adolescents with diabetes; 3) frequency of delivery of education in different topics related to exercise and diabetes; 4) confidence to give information about exercise; 5) application of the 2018 ISPAD Clinical Practice Consensus Guidelines on exercise and children and adolescents with diabetes; 6) training received about exercise and diabetes management; 7) barriers for HCPs to share education about exercise and diabetes and barriers of children and adolescents with diabetes to do exercise, and 8) perceived knowledge of topics related to exercise and diabetes.

The main focus of this research was to develop a greater understanding of the overall level of HCP knowledge and confidence in exercise management in youth with T1D. Survey responses for questions with Likert scales were transformed into a binary variable (yes for responses 4-5 and no for responses 1–3). Survey data were analyzed using Welch's *t*-tests and Pearson's Chi-squared tests and summary data are reported as mean ± standard deviation or median and interquartile range (IQR). Significance was set at *p* < 0.05. All statistical analyses were conducted using R 3.6.1 (The R Foundation for Statistical Computing).

## 3. Results

A total of 125 pediatric HCPs with a median age of 42 (IQR: 26–70) years, 74% female, 47% ISPAD members, and 73% physicians/clinicians from 31 different countries working with children and adolescents with T1D completed the online survey (Table 1). Surveys were completed by HCPs from North America (26%), Central and South America, (11%), Europe (55%), and Other (7%). Respondents that were not actively working with children and adolescents with T1D or that were not providing exercise education to patients (*n* = 3) were excluded from the analysis.

**Figure 1:**
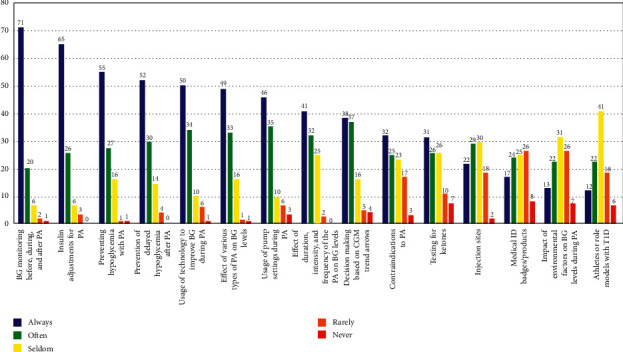
Type and frequency of various exercise-related topics that HCPs discuss with their patients with T1D. Likert scale values included 1 = always, 2 = often, 3 = seldom, 4 = rarely, and 5 = never. Data reported as percentages (%). Note: values may not add up exactly to 100% due to rounding. BG = blood glucose; PA = physical activity; CGM = continuous glucose monitoring.

**Figure 2:**
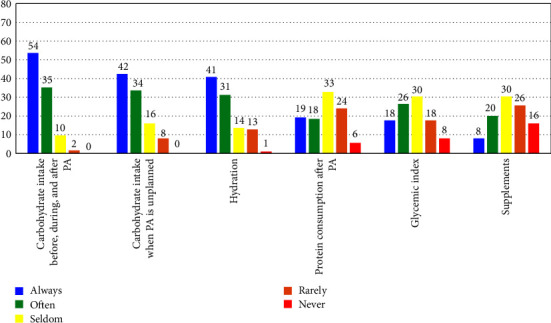
Type and frequency of various nutrition-related topics that HCPs discuss with their patients with T1D. Likert scale values included 1 = always, 2 = often, 3 = seldom, 4 = rarely, and 5 = never. Data reported as percentages (%). Note: values may not add up exactly to 100% due to rounding. PA = physical activity.

The type and frequency of various exercise and nutrition-related topics that HCPs discussed with their patients with T1D are presented in Figures 1 and 2. As noted above, these questions were scored on a Likert scale (i.e., 1–5) and the values represented the following: 1 = always, 2 = often, 3 = seldom, 4 = rarely, and 5 = never. The topics most often discussed by HCPs included blood glucose monitoring before, during, and after exercise; insulin adjustments for exercise; and preventing hypoglycemia with exercise (Figure 1). The topics least discussed by HCPs included specific information about supplements; glycemic index; and medical ID badges or other products for exercise (Figures 1 and 2).

No significant differences in exercise recommendations or familiarity with the ISPAD exercise guidelines were observed by age, sex, type of HCP, years in practice, healthcare type, number of patients seen monthly, or HCP team size. However, significant geographical differences were observed in exercise confidence, as described below (*p* = 0.048, Table 2).

### 3.1. Exercise Recommendations and Encouragement to Engage in Physical Activity

Of the 125 completed surveys, 47/125 (38%) of HCPs recommended that patients aim to achieve between 45–60 minutes of exercise per day, with only 16/125 (13%) recommending 60–90 minutes of exercise per day, with the latter being more consistent with the current ISPAD consensus guidelines on exercise management [6].

### 3.2. Confidence to Educate about Diabetes and Exercise

HCPs were asked how confident they feel about providing exercise-specific information to patients (Figures 1 and 2 include topics discussed) on a Likert scale of 1 to 5 (1 being “not confident” and 5 “entirely confident”). A total of 75/125 (60%) of HCPs felt “very confident” and 16/125 (13%) felt “extremely confident” in giving recommendations about exercise to children and adolescents with T1D (Table 3). A total of 2/125 (1%) HCPs felt “neither confident nor not confident” and 32/125 (26%) felt “somewhat confident” in giving recommendations about exercise to children and adolescents with T1D (Table 3). When transformed into a binary variable, a total of 91/125 (73%) HCPs felt confident and 27% did not feel confident with giving recommendations about exercise (Table 2).

HCPs shared about some areas of exercise and T1D that they believe are lacking resources/information such as motivation to engage in PA, management of glycemia during exercise with hybrid closed-loop systems, management of glycemia during ultra-endurance events or high-level sports, and prevention strategies for delayed exercise-associated hypoglycemia.

A total of 55% of survey respondents reported having received specific “professional training” around diabetes and exercise primarily from endocrinology/diabetes conferences (77%), articles and books (72%), advice from other HCPs (52%), and/or courses or modules (43%). There was a difference in the confidence about diabetes and exercise recommendations depending on the HCPs geographical location. In general, HCPs from Europe expressed significantly higher confidence around exercise recommendations as compared to the other continents (*p* = 0.048) (Table 2).

### 3.3. Barriers for HCPs around Exercise and T1D

HCPs reported a number of barriers around exercise education delivery. These barriers included a lack of sufficient knowledge and/or training in advising youth with T1D regarding exercise (75%); lack of time (71%), lack of resources (41%), lack of interest by children and adolescents (31%), and lack of interest by HCPs (26%).

HCPs considered the main barriers for youth with T1D to exercise to be a lack of motivation (78%), fear of hypoglycemia (57%), lack of knowledge about the importance of exercise and exercise management in diabetes (38%), and lack of access to safe exercise spaces (28%).

### 3.4. ISPAD Exercise Guidelines Familiarity

In addition, HCPs were asked about their familiarity with 2018 ISPAD guidelines on exercise on a scale of 1 to 5 (1 being “not aware of” and 5 “extremely familiar”). A total of 47/125 (38%) reported being very familiar (i.e., 4) with the ISPAD exercise guidelines, and 21/125 (17%) reported being extremely familiar (i.e., 5) with the guidelines (Table 3). When transformed into a binary variable (Table 2), a total of 68/125 (54%) HCPs reported being familiar with the ISPAD exercise guidelines.

## 4. Discussion

In this study, an international survey was used to explore HCPs knowledge and confidence in discussing and delivering exercise guidelines to youth with T1D. In summary, HCPs are often not providing thorough and adequate education and recommendations around exercise for children and adolescents with T1D. In particular, the global recommendation that youth should aim to achieve at least 60 minutes of moderate-to-vigorous-intensity PA per day [6, 8], was “prescribed” by only 13% of the respondents. Surprisingly, a larger percentage of respondents (38%) recommended 30–45 minutes per day of PA, which does have health benefits over less activity, but is more in line with the recommendations for adults [8]. This may be for practical reasons since youth with diabetes tend to be less active than their peers without diabetes [18], or because the recommendations for youth are not well known by the HCP community. In contrast, 73% of the respondents felt confident about sharing information about exercise and T1D with their patients. This suggests that content knowledge of exercise management is reaching HCPs, and they are indeed engaging on the topic with their patients, but that this content knowledge may need to be refined somewhat (i.e., refinement of daily PA recommendation level) and perhaps better reinforced for those youth who are not engaging in enough PA, perhaps because of some of the barriers to PA, also identified in this survey.

All routine clinical practice should include recommendations of regular PA (at least 60 minutes of moderate-to-vigorous PA) for youth with T1D as a core plan to optimize glycemic management and well-being [19]. Additionally, HCPs should provide individualized examples and guidance for youth with T1D diabetes on how regular PA and personal goals can be met [1, 19]. However, uptake of these recommendations from the healthcare team might be challenging, and in some cases, scarce. Therefore, specific strategies to improve the frequency and quality of exercise education in diabetes clinics should be strongly encouraged. Utilizing resources such as the “Moving Medicine conversation templates” can improve and facilitate the conversations about PA between HCP and youth with T1D [19]. It is important to ensure that all HCPs (e.g., nurses, Certified Diabetes Care and Education Specialists, dietitians, physicians, exercise physiologists, etc.) are up to date with the latest evidence-based guidelines around glycemic management in exercise, with a better emphasis on how much activity should be targeted daily [6].

Developing and providing structured and geographically accessible educational materials for managing glycemia around exercise can be a potential strategy to help reduce some barriers and provide support for HCPs and youth with T1D [1]. Peer support group education has also been shown to reduce barriers and increase engagement [20, 21]. Developing structured education programs from the onset of T1D diagnosis is also an innovative and effective way to increase confidence and management strategies for exercise [22]. It may be ideal to implement more formal education around exercise and T1D in the HCP, particularly if it were implemented earlier during training [23]. Some programs (e.g., in the UK the Exercise for Type 1 Diabetes [EXTOD] program) and in North America (e.g., The Endocrine Society Fellows Series) look to deliver structured education to HCPs; however, these programs are not delivered as part of a career pathway, nor do they reach enough HCPs globally with their current platform.

Lack of knowledge was noted by HCPs as the main barrier to exercise counseling, followed by a lack of time. Although most of the respondents reported that they were familiar with the 2018 ISPAD guidelines on PA, many appeared to want more professional education on this topic. Importantly, a majority of respondents claimed that they did discuss several key elements of the guidelines including how exercise type, intensity, and duration can impact glycemia and the importance of glucose and ketone monitoring, technologies, and various hypoglycemia prevention strategies. The use of webinars and/or courses focused on diabetes and exercise along with the creation of more practical exercise management guidelines, in various languages that could also be provided to youth with T1D and their families, could be used as an additional educational support tool. These more “translational” guidelines that could summarize key points and strategies, as well as reinforce the daily and weekly activity goals for patients, may also help overcome the lack of motivation from people with diabetes which was considered a major barrier for individuals with T1D to adhere to PA in their routine.

This study has some limitations that should be noted. First, we acknowledge a possible selection bias of responders as participants were approached through ISPAD channels, expecting them to be familiar with the ISPAD guidelines and therefore, limiting generalizability. Those not involved with ISPAD would likely have less content knowledge of the ISPAD exercise consensus guidelines, albeit they may be well versed on exercise management in diabetes via other means. We noted that slightly more than half of the responders were not ISPAD members, and thus acquiescence bias may have been reduced somewhat. Second, our study, while global, did favour representation from European HCPs (55%), with limited representation from other countries. We did not assess via the survey if the respondents were giving the correct advice around exercise, but rather their perception of knowledge and the confidence to give advice. As such, further research in this area is warranted. Finally, we suspect that the respondents for this survey might be more interested in exercise management, in general, and might over represent some individuals with certain clinical backgrounds, experiences, and training in Europe (i.e., the UK) and North America.

## 5. Conclusion

We conclude that PA counseling, along with safe and effective clinical exercise management recommendations for youth with T1D remains a challenge for HCPs globally. While guidelines recommend at least 60 minutes of moderate-to-vigorous exercise per day, more than half of the responders do not prescribe enough exercise to their patients, although most of them consider themselves informed about the current ISPAD clinical practice guidelines on exercise management for youth with diabetes. Although a majority of HCPs appear to engage with their patients on topics related to exercise management, most would like more content knowledge on this important topic and more time to deliver this content knowledge to their patients.

## Figures and Tables

**Table 1:** Baseline characteristics.

	Overall (*N* = 125)
Sex	
Male	31 (24.8%)
Female	93 (74.4%)
Prefer not to say	1 (0.8%)

Age (years)	
Mean (SD)	44 ± 9
Median (IQR)	42 (26, 70)

Continent	
North America	33 (26.4%)
Central & South America	14 (11.2%)
Europe	69 (55.2%)
Other	9 (7.2%)

ISPAD member	
Yes	59 (47.2%)
No	66 (52.8%)

Years in practice	
Mean (SD)	12 ± 8
Median (IQR)	10 (1, 35)
Missing data	1 (0.8%)

HCP type	
Physician/Clinician	91 (72.8%)
Nurse	15 (12.0%)
Dietitian	15 (12.0%)
Psychologist	4 (3.2%)

Healthcare type	
Public	77 (61.6%)
Private	14 (11.2%)
Both	34 (27.2%)

# Patients seen monthly	
<10	16 (12.8%)
10–25	29 (23.2%)
25–50	39 (31.2%)
50–100	30 (24.0%)
>100	11 (8.8%)

HCP team size	
0–10	90 (72.0%)
11–20	22 (17.6%)
30–50	13 (10.4%)

Data reported as mean ± SD or median (interquartile range). North America: Mexico, Puerto Rico, and the United States. Central and South America: Costa Rica, Panama, Argentina, Brazil, Paraguay, and Chile. Europe: United Kingdom, Turkey, The Netherlands, Sweden, Spain, Slovenia, Portugal, Poland, Lithuania, Italy, Greece, Germany, and Austria. Other: Egypt, the Republic of Congo, India, Taiwan, Australia, and New Zealand.

**Table 2:** Overall survey results (*n* = 125 respondents).

	Exercise recommendations (mins)		Exercise confidence		ISPAD familiarity	
	No (*n* = 61)	Yes (*n* = 63)	No (*n* = 34)	Yes (*n* = 91)	No (*n* = 57)	Yes (*n* = 68)
Sex						
Male	13 (21.3%)	18 (28.6%)	7 (20.6%)	24 (26.4%)	13 (22.8%)	18 (26.5%)
Female	43 (78.7%)	44 (69.8%)	27 (79.4%)	66 (72.5%)	43 (75.4%)	50 (73.5%)
Prefer not to say	0 (0%)	1 (1.6%)	0 (0%)	1 (1.1%)	1 (1.8%)	0 (0%)

Age (years)						
Mean (SD)	43 ± 9	44 ± 10	45 ± 9	44 ± 10	45 ± 9	43 ± 10
Median (IQR)	41 (26, 62)	42 (26, 70)	44 (26, 64)	41 (26, 70)	43 (28, 64)	42 (26, 70)

Continent						
North America	15 (24.6%)	17 (27.0%)	14 (41.2%)	19 (20.9%)	15 (26.3%)	18 (26.5%)
Central & South America	8 (13.1%)	6 (9.5%)	1 (2.9%)	13 (14.3%)	3 (5.3%)	11 (16.2%)
Europe	32 (52.5%)	37 (58.7%)	18 (52.9%)	51 (56.0%)	35 (61.4%)	34 (50.0%)
Other	6 (9.8%)	3 (4.8%)	1 (2.9%)	8 (8.8%)	4 (7.0%)	5 (7.4%)

Years in practice						
Mean (SD)	11 ± 7	12 ± 9	12 ± 9	11 ± 8	11 ± 8	12 ± 8
Median (IQR)	9 (1, 30)	10 (1, 35)	10 (1, 35)	9 (1, 35)	9 (1, 35)	10 (1, 35)
Missing data	1 (1.6%)	0 (0%)	0 (0%)	1 (1.1%)	0 (0%)	1 (1.5%)

HCP type						
Doctor/Clinician	42 (68.9%)	49 (77.8%)	22 (64.7%)	69 (75.8%)	40 (70.2%)	51 (75.0%)
Nurse	8 (13.1%)	7 (11.1%)	4 (11.8%)	11 (12.1%)	6 (10.5%)	9 (13.2%)
Dietitian	7 (11.5%)	7 (11.1%)	5 (14.7%)	10 (11.0%)	8 (14.0%)	7 (10.3%)
Psychologist	4 (6.6%)	0 (0%)	3 (8.8%)	1 (1.1%)	3 (5.3%)	1 (1.5%)

Healthcare type						
Public	37 (60.7%)	40 (63.5%)	19 (55.9%)	58 (63.7%)	37 (64.9%)	40 (58.8%)
Private	7 (11.5%)	7 (11.1%)	2 (5.9%)	12 (13.2%)	4 (7.0%)	10 (14.7%)
Both	17 (27.9%)	16 (25.4%)	13 (38.2%)	21 (23.1%)	16 (28.1%)	18 (26.5%)

# Patients seen monthly						
<10	11 (18.0%)	5 (7.9%)	3 (8.8%)	13 (14.3%)	7 (12.3%)	9 (13.2%)
10–25	16 (26.2%)	12 (19.0%)	10 (29.4%)	19 (20.9%)	14 (24.6%)	15 (22.1%)
25–50	15 (24.6%)	24 (38.1%)	8 (23.5%)	31 (34.1%)	18 (31.6%)	21 (30.9%)
50–100	13 (21.3%)	17 (27.0%)	9 (26.5%)	21 (23.1%)	12 (21.1%)	18 (26.5%)
>100	6 (9.8%)	5 (7.9%)	4 (11.8%)	7 (7.7%)	6 (10.5%)	5 (7.4%)

HCP team size						
0–10	47 (77.0%)	43 (68.3%)	20 (58.8%)	70 (76.9%)	39 (68.4%)	51 (75.0%)
11–20	9 (14.8%)	13 (20.6%)	8 (23.5%)	14 (15.4%)	10 (17.5%)	12 (17.6%)
30–50	5 (8.2%)	7 (11.1%)	6 (17.6%)	7 (7.7%)	8 (14.0%)	5 (7.4%)

Data reported as mean ± SD or median (interquartile range). A) Exercise recommendations: minutes of exercise that healthcare professionals (HCP) recommend to patients during the clinic. B) Exercise confidence: the confidence of HCP to deliver education about exercise and type 1 diabetes. C) ISPAD familiarity: familiarity that HCP has with ISPAD clinical consensus guidelines 2018: exercise in children and adolescents with diabetes.

**Table 3:** Raw survey scores for HCP exercise recommendations (minutes), confidence, and familiarity with the ISPAD exercise guidelines.

	Overall (*N* = 125)
HCP exercise recommendations minutes, *n* (%)	
Less than 15	1 (0.8%)
15–30	13 (10.4%)
30–45	47 (37.8%)
45–60	47 (37.8%)
60–90	16 (12.8%)
More than 90	0 (0%)
Did not answer	1 (0.8%)

Exercise management confidence, likert median (IQR)	4 (3, 4)
1 (not confident)	0
2	2
3	32
4	75
5 (extremely confident)	16

Familiarity with ISPAD guidelines, likert median (IQR)	4 (3, 4)
1 (not familiar)	10
2	21
3	26
4	47
5 (extremely familiar)	21

HCP = healthcare professional. Data reported as percent and/or median (IQR).

## Data Availability

The data was obtained from the questionnaire provided to participants https://forms.gle/FjkJAkDC5X9hRk77A. Once the current work is published, data will be available on https://www.ispad.org.
